# Lytic Induction Therapy against Epstein–Barr Virus-Associated Malignancies: Past, Present, and Future

**DOI:** 10.3390/cancers12082142

**Published:** 2020-08-02

**Authors:** Stephanie Pei Tung Yiu, Mike Dorothea, Kwai Fung Hui, Alan Kwok Shing Chiang

**Affiliations:** Department of Paediatrics and Adolescent Medicine, Li Ka Shing Faculty of Medicine, The University of Hong Kong, Queen Mary Hospital, Pokfulam, Hong Kong, China; stephanie.pty@gmail.com (S.P.T.Y.); mdor@connect.hku.hk (M.D.); kfhui111@gmail.com (K.F.H.)

**Keywords:** Epstein–Barr virus, lytic induction therapy, endemic Burkitt lymphoma, Hodgkin lymphoma, T-/NK-/B-cell non-Hodgkin lymphoma, nasopharyngeal carcinoma, EBV-associated gastric carcinoma

## Abstract

Epstein–Barr virus (EBV) lytic induction therapy is an emerging virus-targeted therapeutic approach that exploits the presence of EBV in tumor cells to confer specific killing effects against EBV-associated malignancies. Efforts have been made in the past years to uncover the mechanisms of EBV latent-lytic switch and discover different classes of chemical compounds that can reactivate the EBV lytic cycle. Despite the growing list of compounds showing potential to be used in the lytic induction therapy, only a few are being tested in clinical trials, with varying degrees of success. This review will summarize the current knowledge on EBV lytic reactivation, the major hurdles of translating the lytic induction therapy into clinical settings, and highlight some potential strategies in the future development of this therapy for EBV-related lymphoid and epithelial malignancies.

## 1. Introduction

Epstein–Barr virus (EBV) infects more than 90% of adults worldwide. While its primary infection is often asymptomatic, it can manifest as infectious mononucleosis (IM) in adolescents and young adults [1]. EBV is also associated with lymphomas such as endemic Burkitt lymphoma (BL), Hodgkin lymphoma (HL), T-/NK-, and B-cell non-Hodgkin lymphoma as well as epithelial carcinomas, which include undifferentiated nasopharyngeal carcinoma (NPC) and a subset of gastric carcinoma (EBVaGC) [2,3,4]. The biphasic lifecycle of EBV allows it to establish latency subsequent to primary infection in which viral gene expression is limited to those that are responsible for tumorigenesis, apoptosis inhibition, immune evasion, and so on [5]. Owing to the limited choice and the low expression of these viral proteins, it is difficult to target EBV-positive tumor cells specifically. In most cases, treatment against EBV-positive lymphomas is similar to those of EBV-negative lymphomas of the same histology, for example, chemotherapy, radiation, and tumor resection [6]. Therapeutic strategies that target EBV in the associated malignancies can result in highly specific killing effects to the tumor cells, but spare the normal cells from toxic effects.

Occasionally, the latent virus within the infected cells enters into lytic cycle, in which >70 viral proteins are produced [5]. The switch occurs upon the expression of immediate early (IE) proteins, BZLF1 (Zta), and BRLF1 (Rta), which transactivate Zta and Rta promoters (Zp and Rp) and activate the expression of viral genes for viral replication, such as BMRF1, BALF1, and BGLF4, as well as that for production of virions, such as BLLF1 and BFRF3 [7]. The activation of IE proteins and promoters can be achieved through post-translational modification of activators or repressors, modulation of cellular signaling pathways, epigenetic regulation, such as DNA methylation; histone modification; cellular stresses, for example, oxidative stress, hypoxia, autophagy, and inflammation, as well as through modulation of host and viral micro RNAs [8,9,10,11]. Owing to the massive number of viral proteins expressed during the lytic cycle, they may be potentially utilized for EBV-specific therapies. One such therapy is the lytic induction therapy in which EBV is reactivated into the lytic cycle that confers cytotoxicity of antiviral drugs to achieve specific killing effects against EBV-positive cells. Although there were many studies in the past decades studying the lytic induction therapy, only a few were conducted in the setting of clinical trials. This review will summarize the current knowledge on lytic reactivation of EBV, the major hurdles of the lytic induction therapy, and highlight some potential strategies in the future development of this therapy for EBV-associated lymphoid and epithelial malignancies.

## 2. Overview of the Lytic Induction Therapy

**Figure 1:**
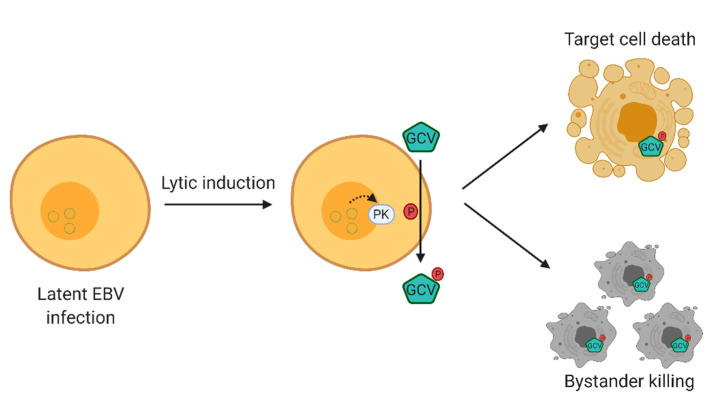
Overview of Epstein–Barr virus (EBV) lytic induction therapy. EBV lytic cycle is first reactivated by chemical inducers in which the viral protein kinase encoded by BGLF4 is produced. BGLF4 then activates the nucleoside analogue antiviral pro-drug into its cytotoxic form, and consequently results in a specific killing effect on EBV-positive cells. Moreover, the activated drugs can be transferred to adjacent cells, resulting in a “bystander killing” effect. GCV, valganciclovir.

Lytic induction therapy is an emerging virus-targeted therapeutic approach that exploits the presence of EBV in tumor cells to confer specific killing effects against EBV-associated malignancies. This strategy involves two classes of compounds, that is, chemical lytic inducers and nucleoside analogue antiviral pro-drugs. The EBV lytic cycle is first being reactivated by the chemical lytic inducers producing an array of lytic proteins, one of which is the viral protein kinase encoded by BGLF4 [7]. This kinase phosphorylates and converts nucleoside analog anti-viral pro-drugs, such as ganciclovir, to their cytotoxic forms, consequently killing their host cells. More importantly, the phosphorylated drugs can be transferred to adjacent cells, which leads to a “bystander killing” effect [12] (refer to Figure 1) [13]. As a result, the success of this method relies heavily on the effectiveness of lytic inducers in reactivating EBV lytic cycle, emphasizing the importance of investigating a broad variety of compounds in order to enable and consolidate this form of therapy for EBV-associated malignancies.

## 3. Lytic Inducers

The lytic induction potential and the modes of lytic reactivation of different compounds, such as histone deacetylase (HDAC) inhibitors, chemotherapeutics agents, phorbol esters, butyrates, and novel compounds, in various cell lines harboring EBV have been summarized in detail in a recent review [14]. Despite having a continuously growing list of lytic inducers that can potentially be incorporated into the lytic induction therapy, very few drugs have been tested in clinical settings [15]. The only clinical trial study to date that has shown a promising outcome tested the effect of combining lytic inducers, gemcitabine (GCb) and valproic acid (VPA), with valganciclovir (GCV) on patients with end-stage NPC [16]. As different classes of lytic inducers have been addressed in detail in other reviews [14,17,18], we will briefly summarize the mechanisms of EBV lytic reactivation and outline the lytic inducers that possess the corresponding reactivation mechanism.

EBV lytic cycle can be reactivated by modulating different signaling pathways of the host, for example, by activating protein kinase C (PKC) directly or together with mitogen-activated protein kinase (MAPK) family consisting of extracellular-signal-regulated kinase (ERK), c-Jun N-terminal kinases (JNK), and p38 signaling pathways [8]. HDAC inhibitors such as suberanilohydroxamic acid (SAHA), romidepsin, valproic acid (VPA), trichostatin A (TSA), and sodium butyrate (NaB) [19,20]; phorbol esters such as tetradecanoylphorbol acetate (TPA) [21]; and microtubule depolymerization compounds such as colchicine and vinblastine [22] have been shown to activate the PKC and/or JNK and p38 signaling to reactivate EBV lytic cycle. TPA activates nuclear factor-κB (NF-κB) and activator protein 1 (AP-1) that mediate the activation of JNK, which may interact with Zp through the binding of c-Jun to the ZI and ZII elements [21]. Another study revealed that Zp activation via PKC-δ activation requires the ZID element, which allows binding of the transcription factor Sp1 [23]. Proteasome inhibitor such as bortezomib and endoplasmic reticulum (ER) stress inducers such as thapsigargin and tunicamycin, on the other hand, can induce EBV lytic cycle by activating ER stress/unfolded protein response (UPR), which induces JNK and/or C/EBP-β and activates Zp through C/EBP-binding sites in ZII and ZIIIB elements [24,25]. UPR-induced lytic reactivation was also observed in clofoctol treatment, which mediates the activation of the PERK-XBP1 axis [26].

Activation of PI3K/Akt signaling pathway can also reactivate EBV lytic cycle. Compounds that possess this property include chemotherapeutic drugs such as gemcitabine, doxorubicin, cis-platinum, and 5-FU [17,27] and immunosuppressive drug such as methotrexate [28]. Phosphoinositide 3-kinases (PI3K) activation was shown to be required for Rta activation of Zp and BMRF1 promoters, albeit the exact mechanism has not been completely elucidated [29]. Immunomodulatory agents such as lenalidomide and thalidomide suppress Ikaros, which can regulate EBV latency as well as activate PI3K signaling [30].

Cellular stress-related signaling pathways involving ATM and p53 can also be associated with reactivation of EBV lytic cycle. Reactive oxygen species (ROS) inducers such as H_2_O_2_, methylnitronitrosoguanidine (MNNG), and the chemotherapeutic drug gemcitabine activate p53, which subsequently binds to the Sp1-binding element in Zp and Rp and activates the lytic cycle [31,32]. Additionally, chloroquine can reactive EBV lytic cycle by chromatin remodeling through the activation of the ATM pathway and the downstream phosphorylation of KAP1/TRIM28 [33], allowing the access of cellular transcription factors to activate the viral promoters [34].

Induction of hypoxia has been shown to reactivate EBV lytic cycle through the binding of hypoxia-inducible factor 1 (HIF-1) to the hypoxia response element motif on Zp and/or by the activation of the ERK1/2 signaling pathway [35,36]. Iron chelators such as deferoxamine, Dp44mT, and a novel compound known as C7 were found to stabilize HIF-1α, which subsequently leads to the reactivation of EBV lytic cycle [36]. Apart from stabilizing HIF-1α, C7 was also found to reactivate EBV lytic cycle through the activation of the ERK1/2-autophagy (ATG5) axis [36]. In addition to C7, autophagy induction through the PKCδ-p38 MAPK axis by combination of TPA and NaB has also been shown to promote EBV lytic cycle [37].

In addition to the above lytic reactivation pathways, other mechanisms such as induction of psychological stress by glucocorticoids such as hydrocortisone and dexamethasone [38], as well as inhibition of NF-κB signaling by antiretroviral medication such as azidothymidine [39], anti-inflammatory drugs or natural compounds such as aspirin [40] and curcuminoids [41], have been found to reactivate EBV lytic cycle. The detailed mechanisms for the reactivation have not been completely delineated. Large-scale screenings of chemical compounds have also identified several novel organic compounds, named E11 and A10 [42], and tetrahydrocarboline derivatives, named C09, C50, C51, C60, and C67, which can induce EBV lytic induction through as yet undetermined mechanisms [43].

## 4. Weaknesses and Concerns Related to the Lytic Inducing Compounds

As mentioned in the previous sections, many efforts have been made in the past years to uncover the mechanisms of chemical compounds in reactivating EBV lytic cycle in both EBV-positive lymphomas and epithelial carcinomas. Despite having the potential of being incorporated into EBV lytic induction therapy regimens, as shown in in vitro testing and Phase I/II clinical trial [16,44], these compounds have major weaknesses in their action. For instance, they have relatively low efficiencies in the reactivation of EBV lytic cycle. Table 1 summarizes the efficiencies of EBV lytic induction by the different compounds from multiple studies. In general, HDAC inhibitors such as NaB could reactivate 2–60% of EBV-positive B cells into lytic cycle, while SAHA could reactivate 30–65% of EBV-associated epithelial cells (AGS-BX1, HA, and HK1-EBV) into lytic cycle [45,46,47]. VPA could induce around 10% of AGS-EBV cells, while the percentage was low in LCL and C666-1 cells [48]. Novel compounds identified by our group such as C7, E11, C8, E7, and A10 could induce 30–60% of AGS-BX1 cells into lytic cycle [42]. Follow-up studies on C7, the best-performing compound identified, showed its ability to induce 6–12% of HA, C666-1, and NPC43 cells into lytic cycle [36,46]. Another new class of compounds, curcuminoids, were shown to induce 20–50% of AGS-BX1, C666-1, and HONE1-EBV cells into lytic cycle [41]. Combination of lytic compounds such as VPA and cisplatin was able to induce 50% of AGS-EBV cells into lytic cycle [48], while 40–70% could be achieved in AGS-BX1, HONE1-EBV, and C666-1 cells treated with VPA together with gemcitabine [41]. The above studies showed that a considerable proportion of cells are refractory to lytic cycle induction by most compounds studied. This refractory population greatly hinders the implementation of these lytic inducers into the lytic induction therapy and the translation to clinical settings.

Second, these lytic compounds rely heavily on the cellular background for inducing EBV lytic cycle. For example, HDAC inhibitor, VPA, could induce EBV lytic cycle in EBV-associated epithelial carcinomas such as C666-1 and AGS-EBV cells [48], but not in EBV-positive lymphomas such as HH514-16, Raji, and Akata cells [49,50]. NaB was shown to induce lytic cycle in EBV-positive lymphoma cell lines including P3HR-1, B95.8, Raji, Daudi [51], and AK2003 [47], as well as in EBV-associated epithelial carcinoma cell line, AGS-BX1 [47], but does so very weakly in NPC cells [41]. SAHA could induce lytic cycle in AGS-BX1, HA [45,46], AK2003 [47], and C666-1 [42] cells, but not in NPC43 [46] and LCLs [47]. Similar results were found in chemotherapy agents such as 5-FU and cis-platinum, which could induce lytic cycle in Akata and AGS-EBV cells [27], but not in LCLs [17]. For other classes of compounds such as tetrahydrocarboline derivatives [43], curcuminoids [41], iron chelators [36], and the novel compounds [36,41,43], lytic induction studies were only examined in either EBV-positive lymphoma cells or a subset of EBV-associated epithelial carcinoma cell lines, thus limiting general conclusions on their abilities to reactivate EBV lytic cycle in both cell types (Table 1). None of the compounds studied to date could induce EBV lytic cycle in all EBV-positive cell lines and their action dependent on cellular background and EBV latency states greatly hinder the incorporation of the available inducers in clinically relevant lytic induction therapeutic regimens.

Lastly, the concern of promoting viral dissemination through chemical induction of EBV lytic cycle has to be addressed with caution. Most of the chemical compounds studied reactivate a complete EBV lytic cycle with production of virions. For instance, supernatant from HONE1-EBV cells induced with SAHA could transduce 71% of Daudi cells in an EBV transduction assay [45]. This raises the concern of promoting viral dissemination in the midst of the therapy. Indeed, a pilot study on the efficacy and safety of romidepsin in treating extranodal natural killer/T-cell lymphoma found a substantial increase in viremia in these patients [52]. The novel compound C7 [36,42,46] and anti-bacterial antibiotic, clofoctol [26], were found to induce the expression of immediately early and early lytic proteins, but not late lytic proteins. Moreover, EBV virions were not produced after lytic induction by these two compounds. The reactivation of EBV lytic cyle without production of virions puts them as potentially suitable candidates for incorporation in lytic induction therapy with minimal risk of viral dissemination.

All of the previously studied compounds have at least one of the three major weaknesses mentioned above. For instance, HDAC inhibitors appear to be efficient in reactivating 30–50% of the cell population into EBV lytic cycle in both EBV-positive lymphoma and epithelial carcinoma cells, but their induction of full viral lytic cycle raises concerns in promoting EBV dissemination during the therapy. On the other hand, C7 and clofoctol are able to induce EBV lytic cycle without production of virions, but a relatively low percentage (10–20%) of cells can be induced into lytic cycle. Therefore, efforts such as structural refinements, as demonstrated in studies by Tikhmyanova et al. [43,53] and our group [36], will be important in promoting the utility of these compounds in lytic induction therapy. Apart from refining the currently available lytic inducers, combination of these different classes of compounds, repurposing of other classes of clinically available compounds, or designing novel chemical molecules or peptides can be employed to facilitate the translation of lytic induction therapy for EBV-associated malignancies into the clinics. These strategies will be discussed in detail in Section 5.

## 5. Potential Drugs and Strategies in the Future Development of Lytic Induction Therapy

### 5.1. Combining Currently Available Lytic Inducers for Induction of EBV

**Figure 2:**
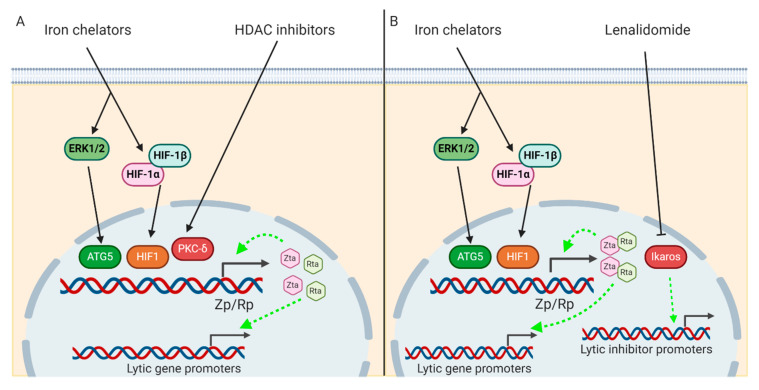
Combination of currently available lytic inducers. (**A**) Combination of iron chelators and HDAC inhibitors. Iron chelators and HDAC inhibitors reactivate EBV lytic cycle through autophagy-dependent and independent pathways, respectively. Their combination could potentially be synergistic in reactivating EBV lytic cycle. (**B**) Combination of iron chelators and lenalidomide. Combination of lytic inducers with different mechanisms for EBV lytic reactivation may have synergistic effects in reactivating the EBV lytic cycle. Lenalidomide reactivates EBV lytic cycle by suppressing Ikaros, which inhibits the expression of transcription factors that inhibit EBV lytic cycle. Manipulation of Zta expression by iron chelators, together with the suppression of inhibitory factors that prevent Zta transactivation of other lytic genes by lenalidomide, may provide a feed-forward loop for lytic reactivation, thus enhancing EBV lytic induction.

Different lytic inducers have been combined in previous studies for reactivating lytic cycle of EBV. The combination between TPA and NaB was found to enhance EA-D expression by 1.5–15-fold more than that by either compound alone in Raji cells [55]. Combination of VPA with cisplatin could induce 50% of AGS-EBV cells into lytic cycle with 1.5–5-fold increase relative to treatment with either compound alone [48]. Additionally, when lenalidomide was combined with doxorubicin or melphalan, lytic induction was enhanced in Daudi and Mutu-I cells [30]. These studies showed that combining different classes of lytic inducers with divergent modes of action in lytic reactivation of EBV could complement one another and achieve a higher efficiency in the induction of lytic cycle of EBV. Iron chelators and SAHA could reactivate EBV lytic cycle by stabilizing HIF-1α [36,56] and activating the PKC-δ pathway, respectively [47]. Our group showed that iron chelators could reactivate the lytic cycle through autophagy-dependent pathways, while SAHA’s action was independent of autophagy [46]. These two compounds might synergize with one another in inducing lytic cycle of EBV (Figure 2A). Combining iron chelators with immunomodulatory agent such as lenalidomide will also be of interest. Lenalidomide could reactivate lytic cycle of EBV by suppressing Ikaros [30], which is a transcription factor that was found to upregulate the expression of cellular factors responsible for maintaining EBV latency [57,58]. Direct activation of Zta promoter by iron chelator through HIF-1α binding, together with the suppression of inhibitory factors that prevent Zta transactivation of other lytic genes by lenalidomide, may provide a feed-forward loop for lytic reactivation of EBV (Figure 2B). Some criteria may need to be considered in the design of combination therapy. First, matching the kinetics of lytic induction of different compounds will be important. Our group found that combination of C7 and SAHA could only enhance lytic reactivation when the treatment duration of C7 matched with its reactivation kinetics [46]. Second, compounds of the same class may not utilise the same mode of action in inducing lytic cycle of EBV. VPA antagonized the reactivation of lytic cycle of EBV by other compounds of the same class such as NaB, TSA, AzaCdR, MS-275, apicidin, and SAHA and uniquely enhanced expression of some cellular genes [55]. Similar antagonism was also observed when romidepsin, another HDAC inhibitor thought to have similar action as that of SAHA, was combined with C7 [46]. Therefore, in-depth study should be performed to delineate the modes of action of different lytic inducers before deciding on the combination therapy.

### 5.2. Repurposing Other Classes of Clinically Available Compounds for Lytic Induction of EBV

Many groups have identified new compounds or re-purposed currently available drugs for reactivating lytic cycle of EBV for lytic induction therapy. The following are some other classes of compounds that have not been explored, but have been shown to modulate pathways involved in regulating the latent-lytic switch of EBV.

#### 5.2.1. Modulators of Autophagy

**Figure 3:**
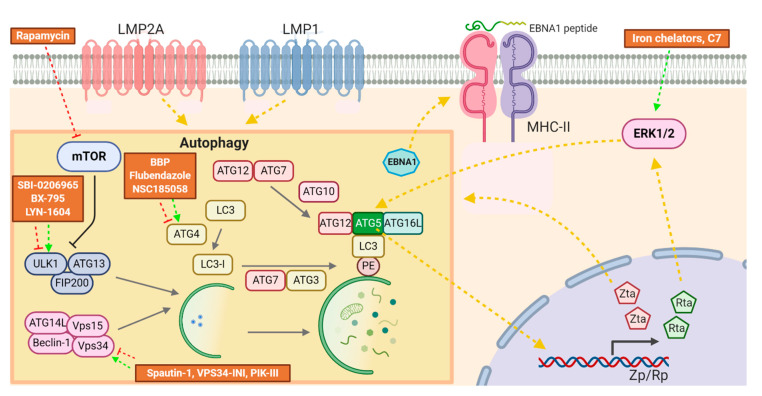
Relationship between induction of lytic cycle of EBV and the autophagy machinery and the modes of action of compounds with lytic induction potentials. EBNA1 could be processed by the autophagy machinery for MHC-II presentation, while LMP1, LMP2A, Rta, and Zta could initiate autophagy. Rapamycin reactivates EBV lytic cycle by inhibiting mTOR. Iron chelators and C7, on the other hand, activate the ERK1/2-ATG5 axis to induce the lytic cycle of EBV. New compounds that target ATG4, ULK1, and Vps34 could potentially reactivate lytic cycle of EBV.

Autophagy is a conserved cellular mechanism that is involved in regulating cellular homeostasis as well as governing cell death and survival. Its progression involves a number of sequential events, that is, vesicle initiation, elongation, maturation, fusion, and degradation, that involve many different tightly regulated autophagic proteins [59,60,61]. Several studies have reported the interaction between the autophagy machinery and EBV latent proteins. For instance, the autophagy machinery processed EBNA1 for its presentation on major histocompatibility complex class II (MHC-II) molecules in EBV-positive B cells [62]. Expression of autophagic proteins was enhanced in B cells and epithelial cells by EBNA3C [63] and LMP2A [64], respectively. Furthermore, LMP1 was shown to initiate autophagy progression in B cells [65,66]. Apart from EBV latent proteins, Rta was shown to initiate autophagy through ERK1/2 signaling pathway. The same study also found that autophagy inhibition by 3-methyladenine (3-MA) or ATG5 knockdown abrogated the expression of EBV lytic proteins and production of virions in B cells [67]. Therefore, modulators of autophagy may represent a potential new class of compounds to be employed for lytic reactivation of EBV. Indeed, our group showed that C7 and iron chelators could reactivate lytic cycle of EBV through autophagy, in particular, through ATG5-related mechanisms [46]. Furthermore, bafilomycin A1, an inhibitor of autophagy, enhanced expression of EBV lytic genes in Akata and Mutu-I cells [68]. On the other hand, a mammalian target of rapamycin (mTOR) inhibitor, rapamycin, which activates autophagy, was found to induce lytic cycle of EBV in EBV-associated epithelial cells [69], but not in B cells [70]. In addition, the effects of other pre-clinical or clinically available modulators of autophagy in the reactivation of lytic cycle of EBV have not been studied in detail. For example, an activator of autophagy, genistein, was shown to have anti-tumor effects in a Phase II clinical trial for prostate cancer [71] and might be a potential compound to be investigated for its role in lytic reactivation of EBV [72,73]. New compounds that have more specific action on particular autophagic proteins such as ULK1, Vps34, and ATG4B have also been developed [74]. Exploring different modulators of autophagy on their effects in inducing lytic cycle of EBV may be relevant for the development of lytic induction therapy (Figure 3).

#### 5.2.2. Modulators of NF-κB Signaling

**Figure 4:**
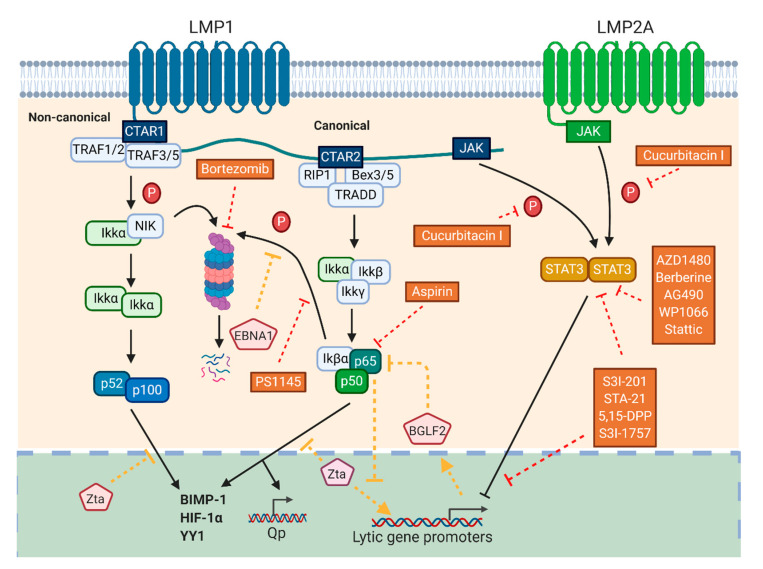
Relationship between EBV proteins, NF-κB, and STAT3 signaling pathways and the modes of action of compounds with lytic induction potentials. LMP1 could activate both canonical and non-canonical NF-κB pathways. RelA(p65) could bind and activate Qp-EBNA1 expression, while itself could, in turn, be inhibited by EBNA1 through the prevention of IκK phosphorylation. RelA(p65) interacts with Zta and abrogates its ability to transactivate other EBV genes, while Zta inhibits the activation of NF-κB-responsive gene promoters. EBV lytic protein encoded by BGLF2 was shown to interact with RelA(p65), preventing its phosphorylation and nuclear translocation. Bortezomib, PS1145, and aspirin reactivate EBV lytic cycle by preventing the degradation of NF-κB inhibitor, the phosphorylation of Iκβα, and translocation of RelA(p65), respectively. Both LMP1 and LMP2A could phosphorylate STAT and inhibit the activation of lytic cycle of EBV. STAT inhibitors such as cucurbitacin I, AZD1480, and S3I-201 reactivate lytic cycle of EBV by either inhibiting phosphorylation, homodimerization, DNA binding, or transcriptional activities of STAT3.

NF-κB transcription factors consist of the REL family members, that is, RelA(p65), RelB, c-Rel, p50, and p52, which are involved in regulating the proliferation, differentiation, and survival of lymphoid cells as well as modulating innate and adaptive immune responses [75]. In response to receptor signal transduction, degradation of the NF-κB inhibitor, IκB, results in the translocation of the transcription factor from the cytosol to the nucleus for transcriptional activation of genes including Blimp1 [76], HIF-1α [77], and YY1 [78], which are known transcription factors that have been shown to modulate lytic cycle of EBV. In addition, activation of NF-κB signaling pathway was shown to be involved in the pathogenesis of EBV-associated diseases. For instance, LMP1 was reported to activate both canonical and non-canonical NF-κB signaling pathways [79,80]. RelA(p65) was shown to bind and activate Qp-EBNA1 expression in EBV-associated epithelial cells [81], while EBNA1 could, in turn, inhibit RelA(p65) by preventing IκK phosphorylation [82]. A more complex interaction was found between Zta and the NF-κB pathway. Interaction between RelA(p65) and Zta abrogated the ability of Zta to transactivate other genes [83,84]. At the same time, Zta inhibited the activation of NF-κB-responsive gene promoters [85], including IκB, which normally retains the inactive NF-κB in the cytoplasm. As a result, a high level of NF-κB was observed in the nucleus [86] along with Zta, inhibiting one another. On the other hand, BGLF2 was shown to interact with RelA(p65), preventing its phosphorylation and nuclear translocation [87]. Therefore, inhibiting NF-κB may lead to reactivation of lytic cycle of EBV lytic [88]. Some previously reported that lytic inducers also act by modulating the NF-κB pathways. For example, a proteasome inhibitor, bortezomib, could reactivate lytic cycle of EBV in Akata and RaeI cells [24] and inhibit the activation of the NF-κB pathway by preventing the proteasomal degradation of the NF-κB inhibitor, IκBα [89]. Aspirin was shown to reactivate EBV lytic cycle in B95.8 and Raji cells by inhibiting RelA(p65) translocation to the nucleus [40]. Owing to the complex interactions among NF-κB, its regulated gene products, and EBV proteins such as Zta, detailed investigations of the effects of modulators of NF-κB signaling pathway on the reactivation of lytic cycle of EBV are indicated. Some potential compounds, including a small molecule, known as PS1145, could specifically inhibit IκB phosphorylation and degradation and the subsequent nuclear translocation of NF-κB in NPC cells [90]. It was also reported to induce lytic cycle of EBV in another study [91]. A sesquiterpene lactone, pathenolide, found in medicinal plants such as feverfew could also inhibit NF-κB and activate the expression of Zta and Rta in Raji cells [92]. Therefore, it is of interest to study the effects of modulators of NF-κB signaling pathway in the reactivation of lytic cycle of EBV (Figure 4).

#### 5.2.3. Inhibitors of Signal Transducer and Activator of Transcription 3 (STAT3)

STAT3 is a transcription factor that regulates a number of physiological processes including apoptosis, immune responses, and cell proliferation. Cytokine such as IL-6 or engagement of growth factor receptors mediates the activation of STAT3, which subsequently translocates to the nucleus and activates the transcription of genes that are involved in the aforementioned biological processes [93,94]. STAT3 is also closely related to the function of various EBV proteins. For instance, LMP2A was found to induce the phosphorylation of STAT3 that activates DNMT1 transcription and leads to the loss of PTEN expression, a common phenomenon observed in EBV-associated gastric carcinoma [95]. A positive auto-regulatory loop between LMP1 and STAT activation was reported in NPC cells [96]. A subsequent study showed that LMP1 triggered the NF-κB, AP-1, and STAT signaling pathways in NPC cells [97], while NF-κB, AKT, and STAT3 were activated by LMP1 in B lymphoma cells [98]. STAT3 was constitutively activated in EBV-positive T or NK lymphoma cell lines [99]. Daigle et al. found that STAT3 level was substantially increased in EBV-positive B cells that were refractory to induction of lytic cycle by NaB [100]. Knockdown of STAT3 sensitized BL cells to lytic inducers for reactivation of lytic cycle, while STAT3 inhibition by small molecules, AG490, WP1066, or stattic was found to reactivate lytic cycle of EBV and enhance induction of lytic cycle in EBV-positive BL cells and LCLs by NaB or Aza [101]. Furthermore, icaritin inhibited STAT3 and AKT pathways by downregulating LMP1 expression, which consequently induced EBV lytic gene expression in ENKL cell lines [102]. In contrast, berberine was found to repress the level of EBNA1 by inhibiting EBNA1 promoter Qp. It also inhibits p-STAT3, consequently reducing the expression of EBV lytic genes and production of virions in HONE1 and HK1-EBV cells upon treatment with NaB and TPA [103]. Cucurbitacin I was found to possess anti-proliferative effects in NPC cells by inhibiting STAT3 phosphorylation. However, its effect in reactivation of lytic cycle of EBV was not examined [104]. JAK2/STAT3 inhibitor such as AZD1480 was found to suppress STAT3 without affecting ERK and AKT signaling pathways [105,106]. Other STAT3 inhibitors such as S3I-201 [107], STA-21 [108], 5,15-DPP [109], and S3I-1757 [110], which prevent STAT3 homodimerization, DNA-binding, and transcriptional activities, should be investigated in their effects on reactivation of lytic cycle of EBV (Figure 4).

#### 5.2.4. Inhibitors of hTERT/NOTCH Signaling

EBV proteins promote tumorigenesis through different mechanisms, one of which is by activating the human telomerase reverse transcriptase (hTERT) promoter by LMP1 through NF-κB, MAPK, and ERK1/2 signaling pathways in B cells and through c-MYC in NPC cells [111]. hTERT is the catalytic component of telomerase that can stabilize telomeres, preventing it from shortening after rounds of cell cycles, thus contributing to the immortalization of cells [112]. In addition, hTERT inhibits the expression of BZLF1 through the NOTCH2/BATF pathway [113] and hTERT silencing by shRNA induces lytic cycle of EBV in BL and LCLs [114]. Apart from its potential role in lytic reactivation, inhibiting telomerase itself may also inhibit tumorigenesis. It would be of interest to study the lytic reactivation ability and cytotoxic effects of the available hTERT inhibitors on EBV-positive cancers. A hTERT inhibitor, BIBR1532, is a synthetic non-nucleoside compound that can selectively inhibit telomerase activity [115]. It can also induce senescence in human cancer cells [116] and possess anti-proliferative effects to leukemia cells, but not normal hematopoietic stem cells [117,118]. It can mediate S-phase arrest in LCLs and BL cells and result in apoptosis [119]. However, its effect on lytic reactivation of EBV has not been studied. Cautions have to be taken with the use of hTERT inhibitors as long-term exposure such as 130 days’ treatment of a hTERT inhibitor, MST-312 [120,121,122], was found to cause cell adaptation by overexpression of telomerase in response to the inhibition in breast cancer cells [123]. Moreover, costunolide was shown to diminish hTERT expression as well as EZH2, H3K27me3, and MSH2 levels in glioblastoma cells. Disruption of EZH2 was shown to increase the expression of both EBV lytic and latent genes including LMP1 [124], suggesting that some hTERT inhibitors might cause reveral of the induction of lytic cycle [125,126] and short incubation time with these inhibitors might be warranted.

**Figure 5:**
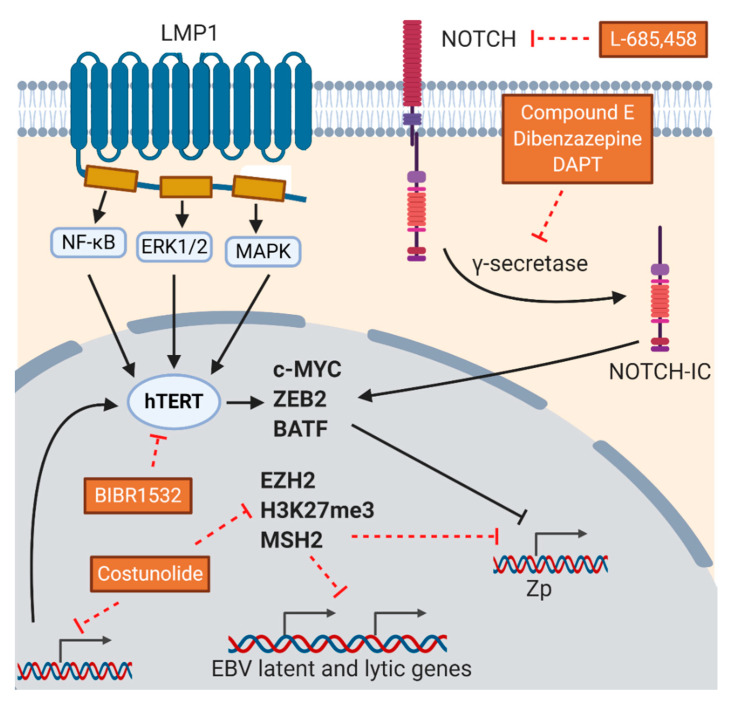
Relationship between EBV proteins, hTERT, and NOTCH signaling pathway and the modes of action of compounds with lytic induction potentials. hTERT inhibits the expression of BZLF1 through the NOTCH2/BATF pathway. EBNA2 is regarded as the functional homolog of active NOTCH intracellular domain (Notch-IC) and LMP2A can activate the NOTCH pathway. NOTCH2 inhibits the reactivation of lytic cycle of EBV through the upregulation of Zeb2 by NOTCH-IC, a transcription factor that represses BZLF1 transcription. γ-secretase inhibitors such as compound E and dibenzazepine can reactivate lytic cycle of EBV by preventing the release of Notch-IC. Other compounds that may reactivate the lytic cycle include hTERT inhibitor, BIBR1532, which selectively inhibits telomerase activity, and another γ-secretase inhibitor, DAPT.

As hTERT inhibits the expression of BZLF1 through the NOTCH2/BATF pathway [113], targeting the NOTCH signaling pathway and examining its effects on induction of lytic cycle of EBV will be of interest. NOTCH receptors are located in the plasma membrane. Upon ligand binding, cleavage on different domains of the NOTCH receptor will occur. The intracellular domain (Notch-IC) released from the transmembrane domain by γ-secretase enters the nucleus and interacts with the transcription factor complex, which consequently activates a number of NOTCH target genes such as MYC and p21 [127]. In the context of EBV, EBNA2 is regarded as the functional homolog of active Notch-IC [128], while LMP2A can activate the NOTCH pathway [129]. Furthermore, activated NOTCH2 was shown to inhibit the reactivation of lytic cycle of EBV through the upregulation of Zeb2, a transcription factor that represses BZLF1 transcription in B cells [130]. Moreover, γ-secretase inhibitors including compound E and dibenzazepine prevented the cleavage of NOTCH2 and inhibited the release of Notch-IC and could transactivate lytic cycle of EBV in LCLs [113]. Another γ-secretase inhibitor, DAPT, may be able to reactivate the lytic cycle of EBV by reducing the amount of cleaved Notch1-IC [129] and the expression of transcription factors involved in endothelial–mesenchymal transition such as ZEB1 and ZEB2 [131]. Indeed, treatment with doxycycline and DAPT in KSHV-infected iSLK.RGB cells were found to increase mRNA expression of viral lytic genes [132]. L-685,458 was also shown to down-regulate c-MYC expression as well as NF-κB and NOTCH pathways in tongue carcinoma cells [133]. It will be of interest to examine the effects of these inhibitors on the reactivation of lytic cycle of EBV (Figure 5).

#### 5.2.5. Inhibitors of MYC

**Figure 6:**
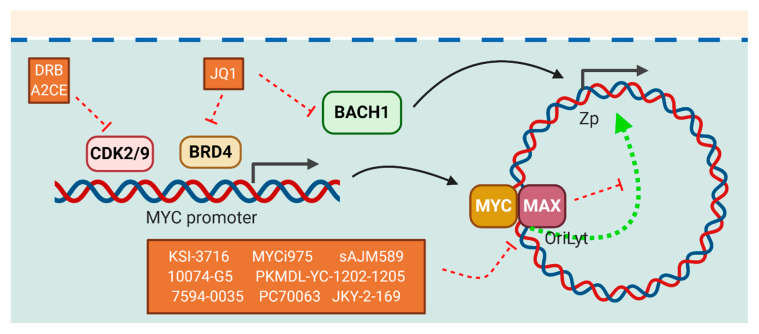
Relationship between EBV proteins and c-MYC and the modes of action of compounds with lytic induction potentials. MYC represses the activation of lytic cycle of EBV by binding to OriLyt on the EBV genome and suppresses its looping to the BZLF1 promoter. A2CE’s and DRB’s inhibition of CDK2/9, a transcription factor that regulates MYC expression, can suppress the expression of EBV latent proteins. Compounds that target the DNA binding domain of the MYC-MAX complex such as KSI-3716, MYCi975, and 7594-0035 may reactivate the lytic cycle of EBV.

MYC regulates many different essential cellular processes including cell proliferation, cell-cycle progression, DNA repair, and survival. Under normal circumstances, MYC expression is tightly regulated and has been shown to be deregulated in over 50% of human cancers [134,135]. In the context of EBV, a recent study on identifying host factors that repress lytic cycle of EBV by a human genome-wide CRISPR-Cas9 screen was performed in BL cells. The identified host repressors were found to be centered on MYC. It was found that MYC bound to the OriLyt on the EBV genome and suppressed its looping to the BZLF1 promoter. Furthermore, depletion of MYC or factors related to MYC expression reactivated the lytic cycle, suggesting that MYC inhibition could reactivate the EBV lytic cycle [136]. Although MYC inhibition would be a direct and powerful approach for the treatment of many types of cancers, MYC lacks a specific active site for binding by small molecules. Therefore, this “undruggable protein structure” greatly hinders the development of chemical compounds inhibiting MYC [137]. As a result, different compounds that indirectly target MYC such as interrupting MYC transcription [138,139], stability [140,141], and the MYC–MAX complex [142] have been developed. MYC transcription is under the regulation of Bromodomain-containing 4 (BRD4) and a BRD4 inhibitor, JQ1, was found to possess anti-tumor effects [138,139]. Of interest, JQ1 was found to inhibit the lytic reactivation of EBV as JQ1 inhibited not only MYC expression, but also other host factors required for activation of lytic cycle including BACH1, whose knockdown reduced the expression of BZLF1 upon treatment with gemcitabine [143]. Similarly, inhibiting CDK7, a transcription factor that regulates MYC expression [144], prevented EBV replication [145], but significantly inhibited cell growth of NPC [146]. Despite the above observations, it was found that DRB’s inhibition of CDK9, another transcription factor that regulates MYC transcription, reduced both MYC and EBNA2-activated transcription [147]. As EBNA2 is the functional homologue of NOTCH that inhibits activation of lytic cycle of EBV, inhibiting EBNA2’s function may reverse the inhibition on lytic cycle. Upon initiation of lytic cycle by transfection of BZLF1 in HEK/EBV cells and incubation with CDK2/CDK9 inhibitor or alsterpaullone 2-cyanoethyl (A2CE), only the expression of late lytic proteins, but not the early lytic proteins was reduced [148]. Other compounds that target the DNA binding domain of the MYC–MAX complex such as KSI-3716 [149], MYCi975 [150], sAJM589 [151], and 10074-G5 [152,153] were found to have anti-tumor effects in multiple tumor cell types. New compounds such as PKUMDL-YC-1202-1205 [154], 7594-0035 [155], VPC70063 [156], and JKY-2-169 [157] have also been developed. It would be of interest to investigate the effects of these compounds on the reactivation of lytic cycle of EBV (Figure 6).

### 5.3. Designing Peptides or Small Chemical Molecules for Lytic Induction of EBV

Protein structural analyses using nuclear magnetic resonance (NMR), cryogenic electron microscopy (Cryo-EM), and crystallography as well as the increasing usage of computer programs in the prediction of active functional domains of protein and docking simulation have accelerated the progress of structure-based drug discovery [154,156,158,159,160]. For example, an EBNA1-specific probe was designed and shown to disrupt EBNA1 oligomerization and transactivation. Furthermore, the probe could reactivate lytic cycle of EBV, indicating that inhibition of repressor of lytic cycle can be harnessed to reactivate lytic cycle of EBV [161,162,163,164]. Hence, cellular factors that were shown previously to inhibit reactivation of lytic cycle can be targeted. Examples are Oct-2 and Pax-5, which are B cell-specific transcription factors [165,166] shown to interact directly with Zta and prevent its binding and transactivation of EBV gene promoters. Furthermore, knockdown of either Oct-2 or Pax-5 could increase the expression of lytic proteins [58,167]. In contrast, the cellular factor, nuclear factor Y (NF-Y), was shown to bind to Rp and the overexpression of NF-YA enhanced the expression of Zta and Rta in NPC. Molecules can be designed to stabilize the binding of NF-Y to the IE promoters, which may, in turn, enhance reactivation of lytic cycle of EBV [168]. Advancement in computational modelling and the resolution of structural interactions between proteins and compounds or peptides can lead to the rational design of highly specific molecules to be incorporated in lytic induction therapy.

## 6. Beyond Lytic Induction Therapy

Apart from lytic induction therapy, identification of essential host factors for the survival of EBV-positive cells can be manipulated to facilitate the development of synthetic lethality. For example, BATF and IRF4 were found to be upregulated upon EBV infection of primary B cells. This resulted in the suppression of BIM and upregulation of MYC, which were found to be important transformation factors for primary B cells upon EBV infection. Knockout of either gene triggered apoptosis of EBV-LCL, suggesting that LCL is addicted to BATF and IRF4 for survival [169]. IRF4 antisense oligonucleotides were found to possess anti-tumor activity in multiple myeloma [170], which was also found to be addicted to IRF4 for survival. Recently, a high-throughput screening of chemical compounds that deplete IRF4 identified several compounds of interest [171] that may be relevant in novel therapeutic approaches against EBV-positive lymphomas. Likewise, the consequences on the host cells upon induction of lytic cycle may potentially be manipulated as therapeutic approaches against EBV-associated cancers. For example, many of the EBV lytic proteins, such as BGLF4 (viral protein kinase), BGLF5 (DNA exonuclease), BALF3 (terminase), and BNRF1 (major tegument protein), were found to induce genome instability [172,173,174,175]. Furthermore, BPLF1 (large tegument protein and deubiquitinating (DUB) enzyme) was found to regulate DNA damage response (DDR) by targeting ubiquitinated proliferating cell nuclear antigen (PCNA). Moreover, overexpression of BPLF1 deubiquitinated PCNA, abolished DDR, and sensitized EBV-positive cells to ultraviolet light and hydroxyurea [176]. Further inhibition of DNA repair mechanism by chemical drugs may overload EBV-positive cells that undergo lytic cycle to DNA damage, thus killing the cells. Advancement of omics technologies may serve to provide an overview of the virus–host interactions and identify host factors that regulate the lytic cycle, which eventually leads to new directions in the design of therapeutic strategies against EBV-associted malignancies.

## 7. Conclusions

In this review, we have summarized the current knowledge of the reactivation of lytic cycle of EBV and the lytic inducers that have been studied in the past decades. We have also addressed the three major weaknesses of the lytic induction therapy, namely, the relatively low efficiency, the high reliance on the cellular background of lytic inducers in the lytic reactivation of EBV, and the concern of viral dissemination during lytic induction therapy. In addition, we have suggested future strategies such as combining different classes of lytic inducing compounds, repurposing other classes of clinically available compounds, or designing novel chemical molecules or peptides to potentiate and translate lytic induction therapy into the clinical settings. Identification of EBV-dependent host factors and proteins involved in the reactivation of lytic cycle will expand our basic understanding of EBV biology and provide valuable insights into the development of new therapeutic approaches against EBV-associated malignancies. 

## Figures and Tables

**Table 1:** Summary of the efficiency of lytic induction of Epstein–Barr virus (EBV) of the lytic inducers and the cell types in which lytic cycle can be induced *.

Class	Compound	Cell Type That Can Be Induced (% of Cell Population)	Cell Type That Cannot Be Induced	Ref.
**HDAC inhibitors**	NaB	HH514-16, B95.8	Raji	[49,50]
		AHS-BX1, BL-AK2003	LCLs	[47]
	TSA	HH514-16, P3J-HR1	Raji, B95.8, Akata	[49,50]
		AHS-BX1, BL-AK2003	LCLs	[47]
	VPA	LCL (low), C666-1 (low), AGS-EBV (10%)	/	[48]
		AGS-BX1	LCLs, BL-AK2003	[47]
	TPA	B95.8, Raji	HH514-16	[49,50]
	SAHA	AGS-BX1, BL-AK2003	LCLs, NPC43	[47]
		HK1-EBV, HONE-1-EBV, HA (30–65%), C666-1	/	[46]
	Romidepsin	HA (75%), C666-1 (6%)	NPC43	[19,46]
**DNA methyltransferase inhibitor**	AZC (5 ara2’-deoxycytidine)	HH514-16	/	[49,50]
		RaeI (80%)	/	[54]
**Iron chelators**	Deferoxamine, Dp44mT	AGS-BX1, SNU719, HA	/	[36]
	Deferasirox, Deferiprone	AGS-BX1, SNU719	/	[36]
**Novel compounds**	C7	AGS-BX1, SNU719, HONE1-EBV, YCCEL-1, HA (10%), C666-1 (6%), NPC43 (12%)	/	[36,46]
	E11	AGS-BX1 (60%), HONE1-EBV, YCCEL-1	SNU719, C666-1	[42]
	C8	AGS-BX1 (30%), HONE1-EBV, C666-1	SNU719, YCCEL-1	[42]
	E7	AGS-BX1 (30%), HONE1-EBV, C666-1, SNU719	YCCEL-1	[42]
	A10	AGS-BX1 (30%), HONE1-EBV, C666-1, YCCEL-1	SNU719	[42]
**Chemotherapeutic agents**	5-FU	Akata, AGS-EBV (24–28%)	LCL	[17,27]
	Gemcitabine	AGS-EBV (30%), Akata, AGS-EBV (24–28%)	LCL	[17,27,48]
	Doxorubicin	LCL [17]	LCL [48]	[17,48]
	Taxol	LCL [17]	LCL [48]	[17,48]
	5 aza-CR	Akata, AGS-EBV (24–28%)	/	[27]
**Immunomodulatory agents**	Lenalidomide, thalidomide, pomalidomide	B95.8, D4 LCL, DAUDI, KEM-I, MUTU-I	/	[17]
**Anti-bacterial antibiotic**	Clofoctol	Akata (40%), SNU719 (2%), C666-1 (0.5%), LCLs (0.5%)	/	[26]
**Curcuminoids**	41	AGS-BX1 (40–60%), C666-1 (10–30%), HONE1-EBV (20–40%)	SNU719	[41]
	EF24	AGS-BX1 (50–70%), C666-1 (10–30%), HONE1-EBV (40–60%)	SNU719	[41]
**Tetrahydrocarboline derivatives**	C09, C50, C53, C60, C67	MutuI, LCL, Akata, C666-1	/	[43]
**ER stress inducers**	Thapsigargin	LCL	/	[25]
**ROS inducer**	N-Methyl-N’-Nitro-N-Nitrosoguanidine (MNNG)	HA, C666-1, NA (70%)	/	[32]

* HDAC, histone deacetylase; NaB, sodium butyrate; TSA, trichostatin A; VPA, valproic acid; TPA, tetradecanoylphorbol acetate; SAHA, suberanilohydroxamic acid; 5 aza-CR, 5-azacytidine; ER, endoplasmic reticulum; ROS, reactive oxygen species.
